# Effects, Feasibility, and Safety of an Early Mobilization Protocol With Immersive Virtual Reality for Dyspnea in Patients With Acutely Decompensated Heart Failure: The MOVE Randomized Clinical Trial

**DOI:** 10.2196/80729

**Published:** 2025-10-31

**Authors:** Iasmin Borges Fraga, Larissa Gussatschenko Caballero, Carlos Eduardo Maciel Tremea, Janaína dos Santos Prates, Gabrielle Perin, Vitor Alves Guedes, Pedro Dal Lago, Eneida Rejane Rabelo-Silva

**Affiliations:** 1 Graduate Program in Cardiology and Cardiovascular Sciences, Faculty of Medicine, Universidade Federal do Rio Grande do Sul, Porto Alegre, Brazil; 2 Heart Failure Clinic, Cardiology Division, Hospital de Clínicas de Porto Alegre, Porto Alegre, Brazil; 3 Graduate Program in Nursing, Nursing School, Universidade Federal do Rio Grande do Sul, Porto Alegre, Brazil; 4 Graduate Program in Rehabilitation Sciences, Physiotherapy School, Universidade Federal de Ciências da Saúde de Porto Alegre, Porto Alegre, Brazil; 5 Physiotherapy Department, Graduate Program in Rehabilitation Sciences, Universidade Federal de Ciências da Saúde de Porto Alegre, Porto Alegre, Brazil; 6 Vascular Access Program, Cardiology Division, Hospital de Clínicas de Porto Alegre, Porto Alegre, Brazil; 7 Graduate Program in Nursing and Graduate Program in Cardiology and Cardiovascular Sciences, Universidade Federal do Rio Grande do Sul, 963 São Manoel Street, Rio Branco, Porto Alegre, 90620-110, Brazil, 55 5133598017, 55 33598657

**Keywords:** heart failure, early mobilization, virtual reality, dyspnea, randomized controlled trial

## Abstract

**Background:**

Early mobilization seems to benefit patients with acutely decompensated heart failure (ADHF), but its initiation is challenging due to severe dyspnea, clinical instability, and low adherence to treatment. Understanding whether early mobilization has good acceptance, adherence, and safety is key for rehabilitation in this population. Virtual reality (VR) offers a less stressful environment and may reduce dyspnea, yet its effects on ADHF remain unknown. In addition, the feasibility and safety of combining VR with an early mobilization program in the intensive care unit (ICU) setting are not fully established.

**Objective:**

This study aimed to assess the effects, feasibility, and safety of an early mobilization protocol combined with immersive VR for dyspnea in patients with ADHF admitted to an ICU.

**Methods:**

The Early Mobilization Protocol with Immersive Virtual Reality (MOVE) study is a single-center, parallel, superiority randomized clinical trial conducted from January 2023 to January 2024 in a teaching hospital in Brazil. Patients with ADHF admitted to the ICU who met the eligibility criteria were invited to participate. After informed consent, participants were electronically randomized into the intervention group (IG) and control group (CG). Both groups underwent up to 3 sessions of the early mobilization protocol supervised by a physiotherapist, including upper- and lower-limb cycle ergometry, standing, and ambulation. Additionally, the IG used VR headsets, headphones, and smartphones displaying 360° videos. The primary outcome was dyspnea measured using the modified Borg scale before and after each session. Secondary outcomes included vital signs before and after each session and the occurrence of adverse events. Physiotherapists were blinded to the primary outcome, and all analyses followed the intention-to-treat principle with a blinded statistician.

**Results:**

A total of 58 participants were randomized (IG: n=28, 48%; CG: n=30, 52%), with a mean age of 59 (SD 11.6) years; 42 (72%) were men, the mean left ventricular ejection fraction was 26.6% (SD 12.5%), and 28 (48%) were categorized as New York Heart Association class III. Only 43% (25/58) of the participants completed all 3 protocol sessions (IG: 11/28, 39%; CG: 14/30, 47%). Reasons for noncompletion of the protocol included refusal, clinical instability, discharge from the unit, or scheduled procedures. Changes in the mean dyspnea scores were similar between groups (IG: −0.17, SD 1.68; CG: 0.01, SD 1.73; *P*=.67). The mean vital signs remained within expected clinical ranges, with no differences between groups (all *P*>.05). No serious adverse events occurred.

**Conclusions:**

Early mobilization with or without VR was feasible and safe in ICU patients with ADHF. VR did not significantly reduce dyspnea, but adherence challenges limited protocol completion. These findings suggest that early mobilization can be safely implemented in this population and that future studies should explore strategies to enhance adherence and evaluate the potential benefits of VR in larger cohorts.

## Introduction

### Background

Patients with heart failure frequently experience periods of decompensation that require hospitalization [1]. Early mobilization has received increasing research attention and has benefits such as shorter hospital stays, lower readmission rates, decreased in-hospital mortality, and improved functional outcomes at discharge [2-4]. Despite these benefits, its early implementation can face considerable challenges due to congestion, respiratory discomfort, multiple medications, and especially intense dyspnea [5,6].

Innovative technologies have been explored as tools to reduce discomfort and promote greater adherence to rehabilitation programs. In this context, virtual reality (VR) stands out as a promising resource that can redirect patients’ attention to a more pleasant environment [7,8].

In patients with advanced heart failure, a 10-minute VR intervention has been shown to benefit the management of self-reported pain, with significantly better outcomes than guided imagery [9]. In other populations, immersive and nonimmersive VR have effectively improved physical fitness and exercise tolerance  [10,11]. However, few studies have included patients with acutely decompensated heart failure (ADHF), and the few available investigations have barely explored the safety and feasibility of these technologies in combination with conventional treatments or their influence on limiting symptoms.

### Objectives

This study aimed to assess the effects, feasibility, and safety of an early mobilization protocol combined with immersive VR on dyspnea in patients with ADHF admitted to the intensive care unit (ICU). The hypothesis tested in this randomized clinical trial was that adding immersive VR to an early mobilization protocol would impact the sensation of dyspnea in patients with ADHF compared to the protocol alone and would constitute a feasible and safe tool.

## Methods

### Study Design

The Early Mobilization Protocol with Immersive Virtual Reality (MOVE) study is a single-center, parallel, superiority randomized clinical trial conducted at Hospital de Clínicas de Porto Alegre (HCPA) from January 2023 to January 2024. The study design and intervention methods have been described previously [12]. This study was registered at ClinicalTrials.gov (NCT05596292), and both the protocol and manuscript were prepared in accordance with the CONSORT (Consolidated Standards of Reporting Trials) checklists (Checklist 1 and Checklist 2) [13].

A pilot study was previously conducted to standardize the mobilization protocol and calibrate the research instruments. Adjustments were made based on the analysis of the pilot data, including adding fields to track participants’ reasons for missing sessions and a descriptive section for session notes, including any adverse events.

### Participants

This study included patients who had been diagnosed with ADHF and had been admitted to the ICU of HCPA. The diagnosis was confirmed by a medical team independent of the research team. Inclusion criteria required participants to be aged more than 18 years, be alert and coherent, have been hospitalized for at least 24 hours, and have received medical clearance to participate in the protocol. Exclusion criteria included invasive mechanical ventilation or circulatory support, neurodegenerative diseases, pregnancy, communication difficulties, or persistent inability to adapt to the VR headset. Patients with severe hemodynamic instability could only be included after clinical improvement (assessed via clinical and imaging examinations). Patients on noninvasive ventilation could participate in the protocol during breaks in ventilatory therapy.

### Intervention and Groups

All eligible patients were registered in an electronic database. Excluded patients, whether for clinical reasons or refusal to participate, had their data collected, including medical record number, reason for exclusion, and date.

After collecting sample characteristics, participants were electronically randomized into the intervention group (IG) or control group (CG). The IG underwent up to 3 consecutive sessions of a progressive and tolerable early mobilization protocol assisted by a physiotherapist combined with immersive VR intervention with headsets compatible with smartphones. A 360° interactive video was displayed during the exercises, enabling patients to explore different angles of the virtual environment. Headphones were used to minimize external sound interference, enhancing the immersive experience. The CG followed the same early mobilization protocol without VR.

The protocol adhered to cardiovascular rehabilitation guidelines, which recommend low-intensity exercise during the acute phase [14]. In total, 3 consecutive sessions were chosen based on evidence suggesting that the most significant changes in dyspnea occur within its first 24 hours, after which changes tend to be less perceptible as treatment progresses [15], potentially compromising the effectiveness of longer protocols.

The protocol included 3 stages of exercise: upper- and lower-limb cycle ergometry, sitting and standing, and ambulation [12]. Exercise duration ranged from 10 to 20 minutes, the progression of which followed individual tolerance (assessed via patient perception and vital signs).

Sessions were conducted in private rooms or isolated hallways to ensure participant privacy. For safety and control, participating patients were identified by a sign on the door of their room indicating their ID number, allocation group, and the start and end dates of the protocol. During the intervention, patients did not participate in any motor activities conducted by the physical therapy team at the hospital.

### Outcomes

#### Primary Outcome

During all sessions, before and after the intervention, the sensation of dyspnea was assessed in both groups using the modified Borg scale [16].

#### Secondary Outcomes

During all sessions, vital signs were recorded before and after the intervention. Serious adverse events, such as dislodgement of venous access or tubes, falls, or signs of hemodynamic instability, were documented.

### Randomization, Allocation, and Blinding

Randomization was conducted at a 1:1 ratio using the REDCap (Research Electronic Data Capture; Vanderbilt University) platform. A member of the research team was responsible for entering patient sample characteristics into the platform, including sociodemographic and clinical data such as New York Heart Association (NYHA) classification, history of hospitalizations due to ADHF, disease etiology, Charlson Comorbidity Index (which estimates 10-year survival), fall risk using the Severo-Almeida-Kuchenbecker scale [17], and functional independence using the Barthel index [18].

These data were used for descriptive analyses and variable control. Patients were randomly allocated to the IG or CG. The physical therapists who carried out the intervention protocol were blinded to the primary outcome, which was collected by independent researchers. To avoid bias, the researchers assessing outcomes withdrew from protocol implementation. Due to the nature of the intervention, patients and physical therapists could not be blinded to group allocation.

### Sample Size, Data Management, and Statistical Analysis

Sample size calculation considered a clinically relevant difference of 1 point in the mean dyspnea sensation between the IG and CG using the modified Borg scale. With a statistical power of 80%, a significance level of 5%, and an SD of 1.6 points (as in the literature) [15,19] and adding 20% for potential losses and refusals, the estimated sample size was 66 participants.

Data were stored on the REDCap platform and analyzed on SPSS (version 28.0 for Windows; IBM Corp). Access to the platform was restricted to trained researchers and team members, and data were extracted for analysis at the end of the protocol. Adverse events, exclusions, and other additional data were documented on the platform. Distribution normality was assessed using the Shapiro-Wilk test. Quantitative variables were described as means and SDs or medians and IQRs depending on their distribution. Categorical variables were described as absolute frequencies and percentages.

All analyses were performed following the intention-to-treat principle. In case of missing data, the means or medians of the allocation group were imputed. To compare means between groups for baseline variables, dyspnea sensation, and vital signs, the Student *t* test (2-tailed) for independent samples was applied. For skewed distributions, the Mann-Whitney *U* test was used as a nonparametric alternative. Proportions between groups were compared using the Pearson chi-square test or Fisher exact test, as appropriate.

The analyses related to dyspnea sensation and vital signs considered 2 assessments: before and after the intervention on the first day and before and after the intervention on the last day of the protocol regardless of whether the patient completed all 3 protocol days. The relative Δ was calculated based on the differences between pre- and postintervention values on each day, whereas total variation was determined using the difference between values on the first and last days of follow-up. To analyze the relationship between vasoactive drugs and dyspnea sensation and vital signs, drugs were grouped into inotropes (milrinone and dobutamine) and vasodilators (nitroprusside and nitroglycerin). Statistical significance was considered as *P*<.05.

### Ethical Considerations

This study was approved by the Institutional Review Board of HCPA (62209822.7.0000.5327) before initiation. Written informed consent was obtained from all participants. For participants unable to read, the consent form was read aloud by the researcher in the presence of a witness. Participation was voluntary, and no financial compensation was provided. Participants’ data were anonymized to protect privacy and analyzed in a blinded manner to maintain confidentiality.

## Results

### Overview

Of the 58 patients (IG: n=28, 48%; CG: n=30, 52%), 25 (43%) completed all 3 sessions of the protocol (IG: 11/28, 39%; CG: 14/30, 47%). In the first session, after consent and randomization, 67% (4/6) of the participants refused the intervention, and 33% (2/6) were excluded due to clinical instability. In the second session of the protocol, 38% (6/16) were discharged from the unit, and the others failed to continue in the study due to clinical instability (4/16, 25%), refusal to continue (2/16, 12%), or other reasons (4/16, 25%). In the third session, 54% (13/24) of the patients were discharged from the unit, 8% (2/24) refused to participate, 12% (3/24) experienced clinical deterioration, 4% (1/24) had a scheduled procedure and were unavailable for the study protocol, and 21% (5/24) were excluded for other reasons.

**Figure 1.**
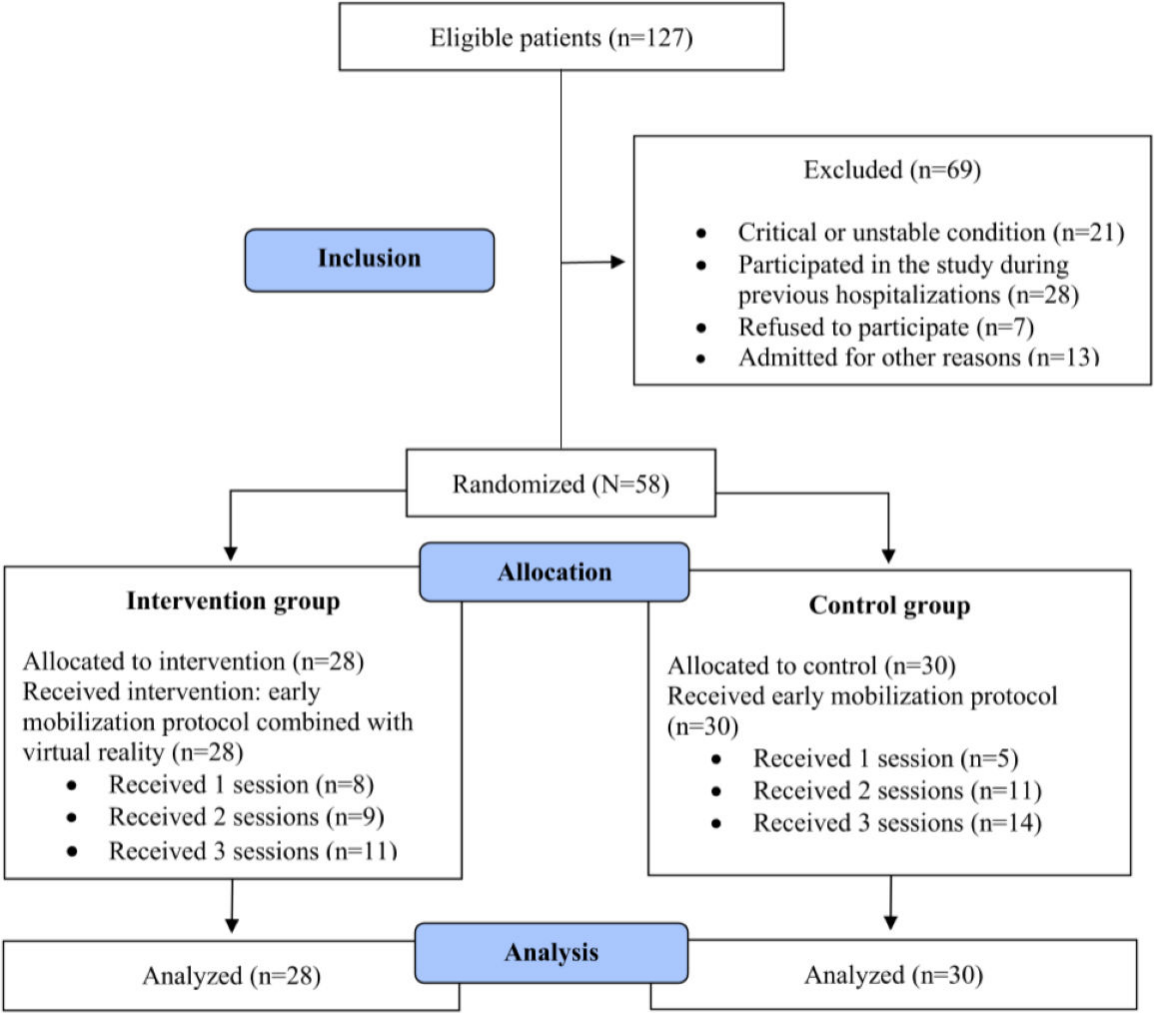
CONSORT (Consolidated Standards of Reporting Trials) diagram of participant progression during the conduct of the randomized clinical trial comparing the intervention and control groups.

“Other reasons” included unit scheduling conflicts, medical team restrictions, and drowsiness. The CONSORT diagram (Figure 1) illustrates the number of patients who completed the protocol.

### Participant Characteristics

The mean age of the participants was 59 (SD 11.6) years, and most were men (42/58, 72%) and had received their diagnosis over 4 years before. The mean left ventricular ejection fraction was 26.6% (SD 12.5%). The etiology of heart failure was predominantly ischemic, and the functional NYHA class at the study inclusion assessment was mostly III. All characteristics between the groups were similar. Table 1 shows these data.

**Table 1.** Participant characteristics (N=58).

Variable	Total	Intervention group (n=28)	Control group (n=30)	*P* value
Age (y), mean (SD)	59 (11.6)	60.7 (10.3)	57.4 (12.7)	.29^a^
Sex, n (%)				.87^b^
Male	42 (72)	20 (71)	22 (73)	
Female	16 (28)	8 (29)	8 (27)	
Weight (kg), mean (SD)	78.3 (22.0)	77.9 (22.8)	78.6 (21.6)	.91^a^
Race, n (%)				.30^c^
Black or of African descent	13 (22)	8 (29)	5 (17)	
Mixed race	4 (7)	3 (11)	1 (3)	
White	41 (71)	17 (61)	24 (80)	
Hospitalizations for HF^d^ in the previous year, median (IQR)	1 (0‐2)	1 (0‐2)	1 (0‐3)	.56^e^
NYHA^f^ classification, n (%)				.90^c^
I	1 (2)	1 (4)	0 (0)	
II	21 (36)	9 (32)	12 (40)	
III	28 (48)	14 (50)	14 (47)	
IV	8 (14)	4 (14)	4 (13)	
Charlson Comorbidity Index (range 0-33 points), median (IQR)	3 (2-5)	3 (2-5)	3 (2‐4.25)	.73^e^
HF etiology, n (%)				.99^c^
Ischemic	26 (45)	12 (43)	14 (47)	
Valvular	6 (10)	3 (11)	3 (10)	
Familial	6 (10)	4 (14)	2 (7)	
Cardiomyopathy	5 (9)	2 (7)	3 (10)	
Myocarditis	5 (9)	2 (7)	3 (10)	
Unknown or idiopathic	5 (9)	3 (11)	2 (7)	
Alcoholic	2 (3)	1 (4)	1 (3)	
Hypertensive	2 (3)	1 (4)	1 (3)	
Cardiotoxicity	1 (2)	0 (0)	1 (3)	
Ejection fraction (%), mean (SD)	26.6 (12.5)	26.1 (11.3)	27.1 (13.7)	.78^a^
SAK^g^ scale, n (%)				.55^b^
Low risk	29 (50)	13 (46)	16 (53)	
Moderate risk	16 (28)	7 (25)	9 (30)	
High risk	13 (22)	8 (29)	5 (17)	
Barthel index, n (%)				.26^c^
Little or no disability	19 (33)	9 (32)	10 (33)	
Moderate disability	31 (53)	13 (46)	18 (60)	
Severe disability	8 (14)	6 (21)	2 (7)	
Vasoactive drugs, n (%)				.19^b^
No	18 (31)	11 (39)	7 (23)	
Yes	40 (69)	17 (61)	26 (87)	
Nitroprusside	28 (48)	11 (39)	17 (57)	.19^b^
Milrinone	18 (31)	8 (29)	10 (33)	.70^b^
Nitroglycerin	6 (10)	4 (14)	2 (7)	.42^c^
Dobutamine	3 (5)	1 (4)	2 (7)	>.99^c^

a Student *t* test.

b Pearson chi-square test.

c Fisher exact test.

d HF: heart failure.

e Mann-Whitney *U* test.

f NYHA: New York Heart Association.

g SAK: Severo-Almeida-Kuchenbecker.

### Dyspnea Sensation

Table 2 shows the variation in dyspnea sensation, expressed using medians and IQRs, between the pre- and postintervention assessments during the first and last sessions of the early mobilization protocol. In the IG, the mean change (Δ) in dyspnea score was lower in the last session than in the first session (0.75, SD 1.91 vs 0.57, SD 1.69). In contrast, in the CG, the variation in dyspnea sensation was similar in both sessions. No statistically significant difference was observed between the groups (difference between groups across the before and after intervention moments on the first day, *P*=.97; difference between groups across the before and after intervention moments on the third day, *P*=.58). The analysis of the difference between the changes (∆ of the last session – Δ of the first session) showed less change in the CG than in the IG, without statistical significance (*P*=.67).

**Table 2.** Variation in dyspnea score between the first and last sessions of the early mobilization protocol in the intervention and control groups (N=58; score range of dyspnea 0-10)^a^.

	Intervention group (n=28)		Control group (n=30)		*P* value
	Variation in dyspnea score, median (IQR)	Δ^b^, mean (SD)	Variation in dyspnea score, median (IQR)	Δ^b^, mean (SD)	
Presession 1	2 (1‐3.75)	0.75 (1.91)	2 (1‐5.25)	0.76 (1.55)	.97^c^
Postsession 1	3 (2-5)	0.75 (1.91)	3.5 (2-7)	0.76 (1.55)	.97^c^
Presession 3	2 (2-4)	0.57 (1.69)	2 (1.75‐4)	0.78 (1.13)	.58^c^
Postsession 3	3 (3-4)	0.57 (1.69)	3.50 (2-5)	0.78 (1.13)	.58^c^

a Δ1 – Δ3 (post–pre between sessions 1 and 3): mean −0.17 (SD 1.68; intervention group) and mean 0.01 (SD 1.73; control group); *P*=.67 (Student *t* test).

b Postsession – presession values.

c Student *t* test.

### Vital Signs

Changes in vital signs, expressed as means and SDs, between pre- and postintervention assessments in the first and last sessions remained within safe ranges for protocol application (Table 3). No differences were observed between the groups.

**Table 3.** Variation in vital signs between the first and last sessions of the early mobilization protocol in the intervention and control groups (N=58).^a^

Variable	Intervention group (n=28), mean (SD)		Control group (n=30), mean (SD)		*P* value
	Before the intervention	After the intervention	Before the intervention	After the intervention	
First session					
SBP^b^ (mm Hg)	101.3 (17.6)	100.6 (18.1)	101.6 (17.6)	107.0 (18.5)	.01
DBP^c^ (mm Hg)	65.5 (11.0)	66.2 (11.6)	65.3 (13.3)	68.8 (12.5)	.22
HR^d^ (bpm)	94.6 (20.5)	95.4 (21.5)	88.9 (15.6)	92.0 (15.7)	.25
RR^e^ (bpm)	18.5 (3.1)	20.3 (4.5)	20.5 (4.4)	20.5 (4.7)	.19
SpO_2_^f^ (%)	96.5 (1.9)	96.8 (1.8)	96.1 (2.4)	96.5 (2.7)	.92
Third session					
SBP (mm Hg)	100.9 (14.3)	105.8 (17.3)	98.7 (11.1)	103.0 (9.9)	.81
DBP (mm Hg)	64.7 (8.8)	67.6 (9.2)	61.7 (6.4)	65.4 (10.0)	.76
HR (bpm)	89.0 (14.2)	90.4 (12.8)	93.7 (14.1)	96.3 (15.3)	.41
RR (bpm)	18.9 (2.9)	20.3 (3.9)	19.0 (3.0)	20.8 (3.8)	.63
SpO_2_ (%)	96.7 (1.4)	97.2 (1.0)	95.7 (2.0)	96.6 (1.4)	.25

a Comparisons between the intervention group and control group were conducted using the mean change (after the intervention – before the intervention) and analyzed using the Student *t* test.

b SBP: systolic blood pressure.

c DBP: diastolic blood pressure.

d HR: heart rate.

e RR: respiratory rate.

f SpO_2_: peripheral oxygen saturation.

The analyses of the last day of the protocol showed a moderate correlation among systolic blood pressure (*r*=0.416; *P*=.03), diastolic blood pressure (*r*=0.393; *P*=.04), respiratory rate (*r*=0.434; *P*=.02), and dyspnea sensation in the IG. In the CG, on the first day of the protocol, a moderate correlation was observed between diastolic blood pressure and dyspnea (*r*=−0.472; *P*=.008). No serious adverse events occurred during this study.

### Stratification by Type of Vasoactive Drug and NYHA Classification

This study grouped vasoactive drugs into 2 categories: inotropes (milrinone and dobutamine) and vasodilators (nitroprusside and nitroglycerin). The intragroup analysis of dyspnea sensation between the first and last sessions of the protocol found no statistically significant differences for any category (*P*>.05). Regarding the functional NYHA class of the patients, neither dyspnea sensation nor vital signs changed in both groups between the first and last sessions (*P*>.05).

### Time Between Hospital Admission and Protocol Start

The median time between hospital admission and the start of the early mobilization protocol was similar between the groups: 4 (IQR 2‐7.7) days in the IG and 4 (IQR 2‐7) days in the CG. In the ICU, patients in the IG took approximately a median of 1.5 (IQR 1‐3.7) days to start the protocol, whereas those in the CG took a median of 2 (IQR 1‐2) days.

## Discussion

### Principal Findings

In this study, an early mobilization protocol with the addition of passive immersive VR failed to significantly change the dyspnea perception of patients with ADHF, although this research considered it feasible and safe. However, the comparison of variations in dyspnea sensation between the first and last day of the protocol suggested that the IG had a smaller change in the perception of this symptom on the last day, whereas the CG maintained similar values over time.

It is also important to highlight that dyspnea, in addition to being related to patients’ clinical condition and pulmonary edema [20], constitutes a subjective symptom that is influenced by previous experiences and patients’ self-management strategies. Those with heart failure, for example, often experience this symptom continuously, which may reduce their change perception of it over time [21]. Furthermore, although various tools can assess dyspnea, the subjectivity of the symptom makes it difficult to accurately identify significant relief, which can compromise the evaluation of therapeutic interventions [22].

VR was applied as an accessible, low-cost, and easy-to-use tool delivered through passive 360° video. Although head movements influenced the viewing angle, there was no active therapeutic goal within the virtual environment. This passive use of VR created a calm and enjoyable setting and has previously been shown to reduce anxiety in other populations, acting as a distraction that can enhance patient cooperation and overall experience, with a low incidence of adverse events such as nausea, vomiting, or dizziness [23]. Both passive and active VR applications have also been shown to be beneficial in pain management [23] and motor rehabilitation [24]. Future studies could explore optimizing 360° video content toward more tranquil and restorative environments, as well as integrating active VR tasks designed to support adherence to clinical interventions. Incorporating real-time feedback on breathing patterns or exercise performance within VR environments could further enhance patient engagement and therapeutic benefit.

Vital signs remained within safe limits, and no serious adverse events occurred in either group, reinforcing the safety of the protocol. Previous studies also indicate that early mobilization protocols are safe for this population, without causing serious adverse events or hemodynamic instability [25]. For the patients who completed the early mobilization protocol, its implementation remained feasible at all stages of treatment. However, data analysis suggests that patients’ short stays in the ICU limited the number of times that the protocol could have been applied. The implementation of the protocol was affected by factors such as the prolonged time that patients spent in the emergency unit waiting for a bed and the team’s uncertainty regarding the initiation of early mobilization. In addition to the cultural barriers commonly faced in early mobilization programs (such as confusion regarding roles, responsibilities, and team attitude), there are patient-related barriers, including limiting symptoms and hemodynamic instability, as well as structural obstacles such as insufficient physical resources and a lack of qualified professionals [26].

These results highlight the feasibility and safety of early mobilization in critical settings and suggest that immersive VR may offer a promising tool to improve engagement and adherence even without objective changes in symptom perception. In addition, future studies are needed to explore the potential of VR in contexts with longer hospital stays or in protocols adapted to patients’ individual preferences.

### Limitations

Less than half of the participants (25/58, 43%) completed all 3 days of the protocol, which limits the generalization of the results. This is partly related to the short stay of patients in the ICU as many experience clinical stabilization while in the emergency department and are then transferred or discharged. Additionally, delays in participant inclusion due to the initial uncertainty of the team also contributed to this outcome. Although the calculated sample size was 27 participants per group, the IG only had 11 participants by the end of the study who had completed the 3 sessions of the protocol. Although all patients who completed at least 1 session of the protocol were included in the analysis, variability in the number of sessions completed and attrition across both groups reduced the effective sample size and, consequently, the statistical power of this study and its ability to detect small to moderate effects. This limitation should be carefully considered in future research. We also highlight the difficulty in recruiting patients from this population, which is characterized by highly individualized clinical conditions and complex management. Thus, while the findings support feasibility and safety, they should be interpreted with caution and are not yet generalizable to all patients with ADHF. Strategies such as recruitment at different stages of hospitalization or across multiple units could extend follow-up and improve the reproducibility of the findings.

### Conclusions

Adding immersive VR to an early mobilization protocol had no significant effect on dyspnea in patients with ADHF. However, the intervention was considered safe and feasible, with no serious adverse events reported in either group.

VR is an accessible and low-cost tool that may have potential applications in outpatient and home settings. Exploring its active use, beyond serving only as a distraction, may provide new insights into its impact on the rehabilitation process in this population. Through facilitating the implementation of early mobilization, VR could contribute to improving clinical outcomes and reducing health care costs. Furthermore, studying the integration of VR into routine care has the potential to optimize rehabilitation guidelines and promote more patient-centered treatment. Future feasibility studies should explore alternative multidisciplinary strategies to enhance adherence and improve the reproducibility of the findings, supporting the translation of this approach into routine clinical practice.

## Supplementary material

10.2196/80729 Checklist 1 CONSORT checklist.

10.2196/80729 Checklist 2 CONSORT-EHEALTH checklist.

## Abbreviations

ADHF: acutely decompensated heart failure
CG: control group
CONSORT: Consolidated Standards of Reporting Trials
CONSORT-EHEALTH: Consolidated Standards of Reporting Trials of Electronic and Mobile Health Applications and Online Telehealth
HCPA: Hospital de Clínicas de Porto Alegre
ICU: intensive care unit
IG: intervention group
MOVE: Early Mobilization Protocol with Immersive Virtual Reality
NYHA: New York Heart Association
REDCap: Research Electronic Data Capture
VR: virtual reality
